# Mechanistic Association of Quantitative Trait Locus with Malate Secretion in Lentil (*Lens culinaris* Medikus) Seedlings under Aluminium Stress

**DOI:** 10.3390/plants10081541

**Published:** 2021-07-28

**Authors:** Chandan Kumar Singh, Dharmendra Singh, Shristi Sharma, Shivani Chandra, Ram Sewak Singh Tomar, Arun Kumar, K. C. Upadhyaya, Madan Pal

**Affiliations:** 1 Division of Genetics, ICAR-Indian Agricultural Research Institute, New Delhi 110012, India; chandankrsingh18@gmail.com (C.K.S.); shristi.sharma1995@gmail.com (S.S.); 2 Amity Institute of Biotechnology, Amity University, Noida 201313, India; schandra4@amity.edu; 3 ICAR-National Institute of Plant Biotechnology, Pusa Campus, New Delhi 110012, India; rsstomar@rediffmail.com; 4 National Phytotron Facility, ICAR-Indian Agricultural Research Institute, New Delhi 110012, India; arun.phytotron@gmail.com; 5 School of Life Sciences, Jawaharlal Nehru University, New Delhi 110067, India; kcupadhyaya@gmail.com; 6 Division of Plant Physiology, Indian Agricultural Research Institute, New Delhi 110012, India

**Keywords:** aluminium, lentil, malate, organic acid, QTL mapping, simple sequence repeats

## Abstract

Aluminium (Al) toxicity acts as a major delimiting factor in the productivity of many crops including lentil. To alleviate its effect, plants have evolved with Al exclusion and inclusion mechanisms. The former involves the exudation of organic acid to restrict the entry of Al^3+^ to the root cells while latter involves detoxification of entered Al^3+^ by organic acids. Al-induced secretion of organic acids from roots is a well-documented mechanism that chelates and neutralizes Al^3+^ toxicity. In this study, F_6_ recombinant inbred lines (RILs) derived from a cross between L-7903 (Al-resistant) and BM-4 (Al-sensitive) were phenotyped to assess variation in secretion levels of malate and was combined with genotypic data obtained from 10 Al-resistance linked simple sequence repeat (SSRs) markers. A major quantitative trait loci (QTL) was mapped for malate (qAlt_ma) secretion with a logarithm of odd (LOD) value of 7.7 and phenotypic variation of 60.2%.Validated SSRs associated with this major QTL will be useful in marker assisted selection programmes for improving Al resistance in lentil.

## 1. Introduction

Aluminium toxicity is amongst the crucial limiting factors which adversely affect growth and productivity. Approximately 40–70% of the cultivable land is affected by Al toxicity worldwide [1]. At low pH (<5.0), Al is solubilized into toxic trivalent cation (Al^3+^) that mainly restricts root growth. This decrease in root length and biomass ultimately reduces whole root system and diminishes subsequent uptake of water and nutrients [2]. Al^3+^ also induces callose formation as a sign of injury, especially at root apex [3].

Two common strategies to alleviate Al toxicity are: soil liming [4,5] and crop breeding to develop resistant genotypes [6,7]. Amongst these, development of Al-resistant genotypes is considered as the most effective, economical and environmentally acceptable strategy to maintain sustainable production under Al toxic soils. To achieve this, it is imperative to know resistance mechanisms in crop plants. Physiological research revealed that plants have evolved two major adaptive mechanisms to cope up with Al toxicity [8]. In the first mechanism, Al ions are restricted to enter the root rhizospheric cells due to secretion of organic acids that chelates and neutralizes Al ions. While, in the second mechanism, inclusion of Al^3+^ ions takes place once they get inside the cytosol, where they bind with organic acids like citrate, malate or oxalate and are transported to vacuoles for detoxification, storage, compartmentalization or immobilization [9,10]. First mechanism further follows two patterns in different plant species based on time period of organic acid(s) secretion from root apex [8]. In pattern I, Al stimulates quick exudation of organic acid(s) within 10–15 min of plant exposure while in Pattern-II, there is a delay phase of about few hours [8]. Transcriptional regulation of genes associated with these mechanisms has also rendered Al resistance in many crops. Genes such as Al-activated malate transporter (ALMT) and multi-drug and toxic compound extrusion (MATE) help in secretion of organic acids under Al stress in wheat [11] and barley [12]. MATE and aluminium-activated citrate transporter 1 (*HvAACT1*), which belongs to the MATE family, were found to be associated with Al-induced citrate secretion in sorghum and barley [13,14]. Higher expression of these genes under Al stress correlated Al^3+^-activated efflux of citrate with Al resistance in these species.

Under Al stress, physio-biochemical and molecular responses have been extensively used as markers to characterize resistant genotypes. Variations in these markers have helped in categorization of genotypes into Al-resistant or Al-sensitive. Efficient screening technique plays an important role to get appropriate differential response in genotypes reflected via stress indicating parameters. In our previous Al stress studies, root re-growth, callose deposition, Al accumulation, antioxidant synthesis and organic acid exudation were efficiently used as identification parameters for Al toxicity resistance [7,15,16,17]. Yusuf et al. [18] assessed Al-resistance of wheat cultivars based on photosynthetic (maximum quantum yield of PSII), osmotic (leaf water potential, electrolyte leakage) and physio-biochemical (lipid peroxidation, hydrogen peroxide production, antioxidant enzyme synthesis and proline content) traits. Similarly, Carcamo et al. [19] tested differential response of blueberry cultivars to Al stress using photosynthetic pigments, antioxidants, total phenols, starch and soluble sugars as marker traits. To date, limited studies have been taken up to decipher physiological and genetic bases of Al-resistance in lentil [7].

The inheritance of Al-resistance can be simple and/or complex in different crops. However, it was found monogenic in lentil [7], pigeon pea [1], pea [20], chickpea [6], bread wheat [21] and barley [22]; whereas, in rice [23], wheat [24] and maize [25], it was of complex pattern. Hence, elucidation of genetics of Al-resistance is a challenging task. Over the decades, selection of Al-resistant genotypes relied upon phenotypic traits, which is environment dependent, time consuming and an exorbitant process [26]. Molecular approaches such as quantitative trait loci (QTL) mapping and genome wide association mapping (GWAS) have made breeding for Al-resistance faster and cost effective [27,28]. Wang et al. [29] developed a high-resolution map of *alp*-locus from a double haploid (DH) population and identified *hvMATE*, a candidate gene for citric acid synthesis in barley. Wang et al. [30] mapped Al tolerance QTLs from soybean F_12_ RILs population by assessing relative root elongation (RRE) and apical Al^3+^ content (AAC) of each line. Ma et al. [23] identified three putative Al tolerance QTLs using RRE trait in backcross isogenic lines (BILs) of rice. Singh et al. [17] mapped Al-resistance loci in lentil using root re-growth after haematoxylin staining and callose accumulation as markers. Organic acid mediated tolerance is considered as a major Al combating mechanism in plants [31].

Lentil (*Lens culinaris* Medikus) is an economically important legume crop and, due to its high nutrition profile, it is consumed and grown worldwide covering a 6.1 million ha area with a production of 6.3 million tons [32].Lentils are rich source of proteins and micronutrients that are grown popularly in India, Canada, Nepal, Syria, China, Turkey, etc. The large cultivable area in these countries has acidic soils with high Al content which leads to the reduced yield of lentils [15]. Being an economically and agriculturally important crop, it is important to understand genetic basis of Al exclusion mechanism as it can help in future breeding programs. No attempts have been made to unravel the genetic basis of Al exclusion mechanism in lentil. To achieve this goal, an efficient screening technique is required which can easily differentiate between Al-resistant and Al-sensitive genotypes. Our previous research in lentil revealed that secretion of citrate and malate was enhanced from roots of both Al-resistant and Al-sensitive genotypes under Al stress condition. However, their secretion was significantly higher in Al-resistant genotypes [16]. Keeping in view the above facts, present study was designed to identify and map QTL(s) associated with malate secretion to induce resistance under Al stress using a RIL population derived from a cross between Al-resistant (L-7903) and Al-sensitive (BM-4) genotypes. Molecular markers (SSRs) linked with these QTL(s) will facilitate marker assisted breeding for improving Al resistance in lentil.

## 2. Results

### 2.1. Phenotyping of Morpho-Physiological Traits in Response to Al Stress

#### 2.1.1. Root Elongation Rate

**Figure 1:**
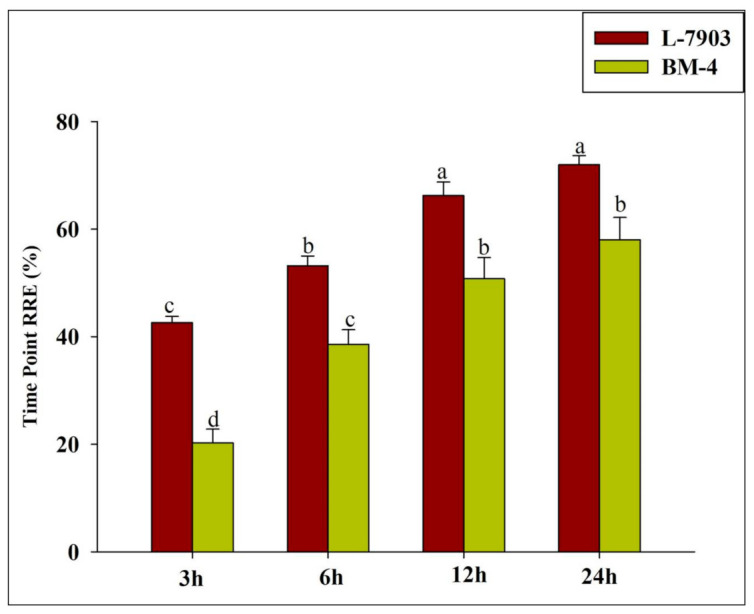
**Relative** root elongation (RRE%) of resistant (L-7903) and sensitive (BM-4) parents under Al stress (148 μM AlCl_3_.6H_2_O) at different time points. Mean values amid same small letters (a,b,c,d) are not statistically different.

Al-resistant genotype (L-7903) showed higher relative root elongation (RRE) as compared to a sensitive one (BM-4) under all the observed time intervals (3, 6, 12 and 24 h). The most significant difference between RRE rate was observed after 3 h in both Al-resistant and Al-sensitive genotypes (Figure 1).

#### 2.1.2. Callose Accumulation in Roots

**Figure 2:**
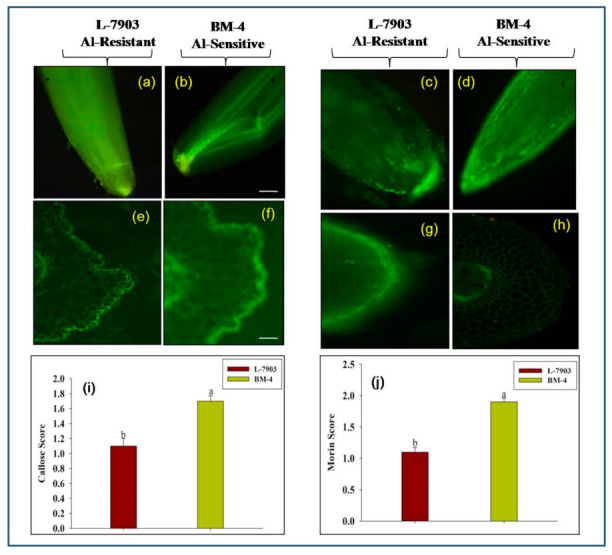
Fluorescent staining assay using aniline blue to detect level of callose in (**a**,**b**) root tips and (**e**,**f**) root cross-sections and morin dye to detect Al localization on (**c**,**d**) root tips and (**g**,**h**) root cross-sections of resistant (L-7903) and sensitive (BM-4) parents.

After 3 h exposure to Al stress, callose accumulation was higher in root tips and root cross-sections of Al-sensitive genotype (BM-4) as compared to Al-resistant one (L-7903). Higher fluorescence intensity was noticed in Al-sensitive genotype with callose score of 1.73 than the Al-resistant one that recorded callose score of 1.07 (Figure 2a,b,e,f,i).

#### 2.1.3. Al Localization in Root Using Morin Assay

Based on morin assay, differential fluorescence intensity was recorded in Al-resistant and Al-sensitive genotypes after 3 h of Al exposure. Al-sensitive (BM-4) genotype revealed higher fluorescence intensity in the epidermis and outer cortex layers with a score of 1.93, whereas the score was less (1.10) in resistant (L-7903) genotype. After same time span, free hand cross-sections of exposed roots showed Al within the epidermis of both the genotypes; however, it was found to be localized in deeper cortical parenchyma layers of Al-sensitive genotype (Figure 2c,d,g,h,j).

#### 2.1.4. Al Content in Roots

Accumulation of Al varied significantly between both the genotypes, where Al-sensitive genotype (BM-4) accumulated higher quantity of Al than Al-resistant one (L-7903) after 3 h of Al-stress. This shows restricted entry of Al^3+^ in the resistant genotype.

### 2.2. Mapping a Major QTL for Secretion of Malate

**Figure 3:**
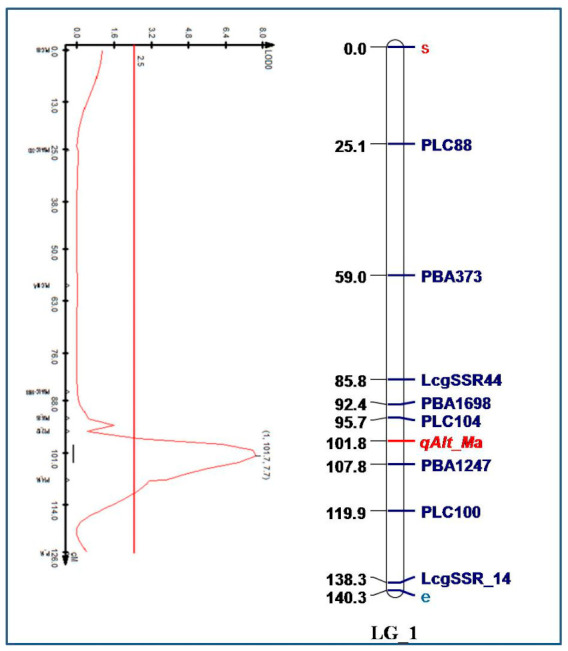
A major quantitative trait loci (QTL) associated with malate secretion under Al stress condition.

Malate was secreted from Al exposed roots of both the parental genotypes. Quantity of malate secretion in Al-resistant (L-7903) and Al-sensitive (BM-4) genotypes were 10.2 and 5.4 nmol h^−1^ g^−1^ fresh weight, respectively. While no organic acid was detected in both the parents without Al stress. Among 146 F_6_ lines of RIL population malate content ranged from 3.8–12.1 nmol h^−1^ g^−1^ fresh weight Plants which secreted malate contents above 8.0 and below 6.0 nmol h^−1^ g^−1^ fresh weights were considered as Al-resistant and Al-sensitive, respectively. Segregation ratio of Al-resistant and Al-sensitive lines in the F_6_ RIL population was 82:64 for malate secretion. This indicated segregation ratio of 1:1 (χ^2^ = 2.22, *p* = 0.136), revealing that malate secretion is controlled by a major gene. QTL analysis estimated linkage between malate secretion and genetic markers (Table 1). When 10 SSRs previously mapped on LG1 harbouring Al resistance QTL [17] were further tested on the RIL mapping population of 146 F_6_ lines, 8 SSRs were again found to be linked, covering a total map distance of 138.3 cM. At a logarithm of odd (LOD) value of 7.7, a major QTL for Al-induced malate (*qAlt*_*ma*) secretion was detected at 101.7 cM on LG1. The identified QTL was flanked by SSR markers viz. PLC 104 and PBA_LC_1247. The observed phenotypic variations explained within RIL population for malate secretion was 60.2% (Figure 3).

## 3. Discussion

QTL analysis was done to locate SSR markers linked with secretion of malate in lentil, which is an exclusive part of exclusion mechanism to tolerate Al stress. Identification and development of Al-resistant genotypes are the best methods to mitigate yield loss due to Al toxicity under acidic soils. Efficient screening technique for parameters that can clearly differentiate Al-resistant and Al-sensitive genotypes is a prerequisite to map QTLs associated with them. Al-resistant (L-7903) and Al-sensitive (BM-4) genotypes were clearly differentiated with the help of parameters—RRE, fluorescent staining assay (using aniline blue and morin)—which estimated callose and Al contents in the roots of plants exposed to Al stress for 3 h. Results revealed that Al-resistant (L-7903) genotype had higher RRE (%) and lower fluorescence. RRE was used as an Al-resistance indicator in barley [29,33], rice [34] and wheat. Aniline blue and morin staining assays to denote Al resistance is also reported in rice [35], sorghum [36] and lentil [16,17]. These results also suggest that 3 h of 148 μM Al exposure is sufficient to screen the genotypes as Al-resistant or Al-sensitive. Further, after same time interval of 3 h, Al-induced malate secretion was highest in lentil roots of both Al-resistant and Al-sensitive genotypes [16]. Therefore, Al exposure period of 3 h is optimum to estimate the quantity of Al-induced organic acid (malate) secretion from roots of Al-resistant and Al-sensitive parents as well as their F_6_ RIL population.

Deleterious effects of Al^3+^ ions are mitigated due to organic acid secretions—which protect the root architecture allowing undisturbed nutrient flow through roots ensuring plant’s proper growth and development. Resistance due to citrate and malate secretion from the roots of Al exposed plants has been reported in crops such as oat [37], barley [22], wheat [38,39] and lentil [16]. Higher exudation of citrate and malate from Al-resistant genotypes in many crops also favours efficiency of this mechanism [40]. Significantly high exudation of organic acids in Al-resistant lentil genotypes than Al-sensitive ones was reported by Singh et al. [16] under different levels of Al stress conditions. They also reported a strong association between higher malate and citrate secretion and lesser Al accumulation in Al-resistant genotypes. However, malate secretion was higher than citrate from the exposed roots of Al-resistant and Al-sensitive genotypes of lentil. Therefore, Al-induced malate secretion in root rhizosphere was quantified and used as a phenotypic marker to differentiate parents and their F_6_ RILs population. The genotypic and phenotypic variations at F_6_ stage of mapping population were integrated to identify and map QTLs using previously reported SSRs for Al tolerance. A major QTL for malate (*qAlt*_*ma*) was mapped on LG 1, with an LOD value of 7.7 and phenotypic variance of 60.2%. The QTL was flanked by PLC_104 and PBA_LC_1247 markers.

Role of organic acid exudation in response to Al stress has been reported in few other legumes such as soybean [41,42] and common bean [43,44]. However, to the best of our knowledge, no study has been conducted to map the gene(s) or QTL(s) associated with malate secretion under Al stress in legumes specially lentil. QTLs related to other Al stress resistance parameters like fluorescence signals and relative root growth (RRG) have been identified in lentil with phenotypic variance of 11.0 and 52.0%, respectively. Seven SSR markers linked with these traits were also mapped on LG1 when F_2_ population derived from a cross between BM-4 X L-4602 was grown under Al stress conditions. The same linkage group was also validated on F_3_ generation of another cross between BM-4 X L-7903 [17]. Further, F_3_ population derived from the cross BM-4 X L-7903 was advanced to F_6_ generation through single seed descent method. The same population has been used in the present study to identify the QTL(s) associated with Al-induced malate secretion. Similar to this study, Al-induced citrate secretion was used as a phenotypic trait in barley to identify and map associated QTLs on 4H chromosome with phenotypic variance of 51.0%. Citrate exudation from root apices of Al-resistant sorghum line was found to be controlled by *AltSB* locus which explained more than 80% phenotypic variance in its mapping population [13,45]. ALMT1 identified in wheat had a role in Al-activated efflux of malate from root apices. This gene was mapped on chromosome 4DL using ‘Chinese Spring’ deletion lines. It was later validated on five different double haploid populations which affirmed governance of Al resistance by single major gene [21]. Genome wide association mapping study involving 1055 accessions and 178 polymorphic diverse arrays technology (DArT) markers have identified various loci for Al resistance including malate efflux in common wheat [46].

Malate synthesis and exudation are controlled by malate dehydrogenase (MDH) and ALMT genes, respectively. Over expression of MDH gene regulated by PrbcS promoter raised malate content that enhanced the Al-resistance in transgenic tobacco [47]. Efflux of malate is mediated by trans membrane protein ALMT [48,49] which is a major contributor of Al resistance in *Arabidopsis thaliana* [50], wheat [51] and other crop plants [48]. In one of our previous study, ALMT-1 gene was also found to be up-regulated in Al-resistant lentil genotypes under Al stress when compared to Al-sensitive ones. Further, sequence alignment of this gene with lentil draft genome located its position on *LcChr*1 and 4 (*Lens culinaris*, chromosomes 1 and 4) with bit scores of 204 and 66.1, respectively (data not published).

## 4. Materials and Methods

### 4.1. Plant Material

To identify Al resistance QTLs associated with organic acid secretion, a F_2_ mapping population derived from the cross between BARI Masur-4 (BM-4, Al-sensitive, high yielding variety form Bangladesh Agricultural Research Institute, Joydebpur, GazipurBangladesh) and L-7903 (Al-resistant, breeding line from Indian Agricultural Research Institute, New Delhi, India) [15,16] was advanced to F_6_ generation (*n* = 146) by single seed descent (SSD) method. Individual F_6_ plants were used in genetic analysis and QTL mapping for Al resistance.

### 4.2. Estimation of Relative Root Elongation in Response to Al stress 

Seeds of both the parents (BM-4 and L-7903) were disinfected by using HgCl_2_ (0.1%) for 3 min, thoroughly rinsed and transferred for germination under control environment condition of growth chamber programmed at 23/18 °C, (±2 °C) day/night temperature and relative humidity of 45%. Thereafter, seedlings (6 days old) were transferred to nutrient medium having composition as described in Singh et al. [16]. After two days of growth in this medium, the seedlings were exposed to Al treatment (148 μM Al, pH = 4.7) for 24 h. A similar set of seedlings were maintained under control condition (0 μM Al, pH = 4.7). Roots of both the genotypes under control and Al-treated conditions were scanned using root scanner (EPSON v.700, Suwa, Nagano, Japan) at time intervals of 3, 6, 12 and 24 h with 10 replicates and the results were analysed using WinRHIZO software (v2013, Regent Instruments, Montreal, QC, Canada). Relative root elongations (%) of both the genotypes at these intervals were deduced following the method of You et al. [52].

### 4.3. Determination of Al-induced Callose Formation 

The seedlings (7 days old) were exposed to control and Al-treated conditions for 3 h as described above. Subsequently, their root tips First 1 cmwere fixed in FAA (10% formaldehyde, 5% glacial acetic acid and 10% ethanol) solution stained with aniline blue (fluorescent dye) and cross sectioned following the method described in Singh et al. [16] and Singh et al. [17], respectively. Fluorescence intensities were observed under a fluorescence microscope (Zeiss, AXIOSKOP 2, Oberkochen, Germany) and their values were scored within 1.0 to 3.0 scale, following the criteria described by Singh et al. [7].

### 4.4. Determination of Al Accumulation using Morin Assay

Accumulation of Al was visualized after 3 h by staining the roots with morin dye which binds specifically with Al to emit green fluorescence [16]. Fluorescence intensity was scored within a range of 1.0 to 3.0 indicating low to high Al accumulation in the roots. To visualize Al localization in different layers of root tissues, free hand cross-sectioning and staining with morin dye were also performed.

### 4.5. Determination of Al Content

Exposed seedling roots under control and Al-treated conditions were excised and oven dried at 65 °C. Dried samples were thoroughly powdered and exposed to a mixture of nitric acid and perchloric acid (3:1 ratio, *v/v*). Al content (mg/g dry weight) was calculated using Atomic Absorption Spectrophotometer (Model 5000, Perkin-Elmer, Shelton, CT, USA).

### 4.6. Estimation of Organic Acids in Roots in Response to Al Stress

**Figure 4:**
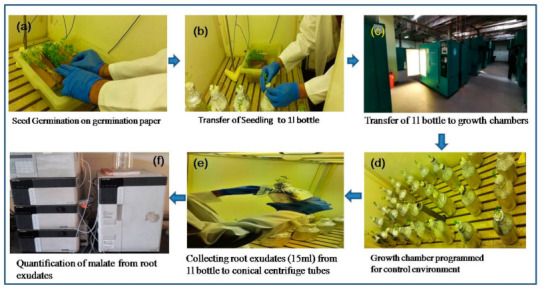
Schematic representation of experimental setup for the estimation of organic acid in growth chamber. (**a**) Seed germination of parents and F_6_ RILs on germination paper, (**b**) Transfer of seedlings to 1l bottle (**c**) Transfer of bottles with seedlings to growth chamber, (**d**) Growth chamber programmed for control environment (**e**) Collection of root exudates from each bottle (**f**) Quantification of malate from root exudates using high performance liquid chromatography (HPLC).

Collection of root exudates was done as per Singh et al. [16]. Twelve individual seedlings of each parent and 146 F_6_ lines were placed in plastic containers (1 L volume) filled with a solution of 0.5 mM CaCl_2_ and 148 μM AlCl_3_.6H_2_O (pH 4.7) in double distilled water. A positive control comprising both the parents that were exposed to0.5 mM CaCl_2_ dissolved in distilled water without any Al stress (pH 4.7) were also included. Root exudates (15 mL) from each container were collected after 3 h of Al exposure along with positive control without Al stress. The experiment was conducted under controlled conditions where the growth chamber was optimized to day/night temperature of 23/18 °C, (±2 °C) with day and night cycle of 10 and 14 h, respectively. Light intensity and relative humidity were maintained at 550 μmol m^−2^ S^−1^ and 45 ± 2%, respectively (Figure 4).

### 4.7. Mapping of Molecular Markers 

Twelve seedlings of the both the parents and plants from 146 F_6_ lines of the RIL population were used for extraction of genomic DNA following Doyle and Doyle [53] method. Quality and quantity of extracted DNA were tested on 1% agarose gel and spectrophotometer (Model 5000, Perkin-Elmer, Shelton, CT-USA), respectively.

Mapping population was genotyped using 10 SSR markers derived from linkage group (LG) 1 as reported by Singh et al. [17]. Polymerase chain reaction (PCR) and its amplification conditions were implied as described in Singh et al. [17]. Genotypic data were combined with organic acids secretion phenotypic data of 146 RIL lines to map Al resistance QTLs.

### 4.8. Construction of Linkage Map

MAPMAKER/QTL version 1.1b (Lander et al. 1987) [54] was used to construct linkage map from 10 SSR markers. Mapmaker software’s Kosambi function [55] was used to deduce map distance along with the locus order. Overall graphical representation of the linkage map was obtained using Map-Chart 2.1 program [56]. QTL cartographer version 2.5 was used for composite interval mapping (CIM) [57]. Threshold logarithm of odd (LOD) ratio for the phenotypic traits under 1000 permutations with 0.05 significance was fixed, with forward–backward stepwise regression. Background controls for different marker co-factors were also fixed.

## 5. Conclusions

Mitigation strategies commonly employed for Al toxicity includes liming and development of Al resistant varieties, where latter is more reliable and gaining momentum. It involves study of Al stress tolerance parameters and their associated genes. Understanding the mechanism of Al toxicity mitigation is important for sustainable agricultural production of lentils and related species. The present study highlighted the use of Al stress indicating parameters such as RRE (%), fluorescent staining techniques like aniline blue and morin for initial differentiation of Al-resistant and Al-sensitive genotypes under hydroponic condition. Al-induced malate secretion was used as a phenotypic trait to identify and map QTL(s) using RILs population derived from cross between BM-4 X L-7903. Association of two flanking SSRs, namely PLC_104 and PBA_LC_1247 with organic acid QTLs, can be used for marker assisted selection and breeding of Al-resistant varieties. Further in these breeding programs, L-7903 can be used as a donor parent for introgression of Al resistance gene. Thereby, QTL identification and marker assisted selection for breeding Al-resistant varieties can surely provide new sources for gene introgression. Moreover, similar strategies can be employed in other legumes for increased production under Al toxicity.

## Figures and Tables

**Table 1:** Malate content in the root exudates of Al-resistant (L-7903) and Al-sensitive (BM-4) parents and their 146 F_6_ lines of recombinant inbred line (RIL) population.

Trait	Parents		RILs (BM-4 X L-7903)		χ^2^	*p*-Value
	L-7903 (Al-Resistant)	BM-4 (Al-Sensitive)	Resistant Lines	Sensitive Lines		
Malate content (nmol h^−1^ g^−1^ FW)	10.2	5.4	8.27–12.1	3.85–6.23	-	-
Number of plants	-	-	82	64	2.22	0.136

## Data Availability

Data sharing is not applicable to this article.
